# Evaluating the expression level of serum Interleukin-2, lipoarabinomannan and circulating MicroRNA-29a as diagnostic biomarkers for pulmonary and extra-pulmonary tuberculosis: a pilot study

**DOI:** 10.1080/07853890.2025.2527949

**Published:** 2025-07-07

**Authors:** Subhamay Adhikary, Sneha Leo, Antara Banerjee, Surajit Pathak, Meenakshi Narasimhan, Sridhar Rathinam

**Affiliations:** a Medical Biotechnology Lab, Faculty of Allied Health Sciences, Chettinad Hospital and Research Institute, Chettinad Academy of Research and Education, Kelambakkam, India; b Department of Respiratory Medicine, Chettinad Hospital and Research Institute (CHRI), Chettinad, Academy of Research and Education (CARE), Kelambakkam, India

**Keywords:** LAM, IL-2, miR-29a, tuberculosis, biomarker, diagnosis

## Abstract

**Background:**

Diagnosis and treatment of Tuberculosis (TB), particularly delay in diagnosis of TB poses significant challenges in its eradication. The exploration of new biomarkers is urgently required for TB diagnosis and treatment. This study aimed to investigate the serum levels of Interleukin-2 (IL-2), an important diagnostic parameter in TB; lipoarabinomannan (LAM), a key constituent of the mycobacterial cell wall; and the expression of circulating microRNA-29a (miR-29a) in serum. MiR-29a contributes to increased susceptibility to TB by downregulating interferon-γ expression in T cells through post-transcriptional regulation, thereby exerting an immunosuppressive effect.

**Materials and methods:**

The study was conducted on pulmonary TB (PTB), extra-pulmonary TB (EPTB), and control groups. Serum from the three groups was isolated, and IL-2 and LAM levels were measured by ELISA. Additionally, q-RT-PCR was conducted to analyse the expression of microRNA-29a in the serum of some TB patients.

**Result:**

LAM and IL-2 were significantly upregulated in serum samples from both the PTB and EPTB groups compared to the control group. Additionally, the expression of miR-29a was significantly elevated in the EPTB group.

**Conclusion:**

This study suggests that LAM and IL-2 may be potential diagnostic biomarkers for both PTB and EPTB, while miR-29a may be a promising marker specifically for EPTB. However, further evaluation with larger cohort samples is required to validate the clinical utility of LAM, IL-2, and miR-29a as diagnostic TB markers.

## KEY MESSAGES

- Serum lipoarabinomannan (LAM), IL-2 and miR-29a might serve as potential markers for TB diagnosis.

## 1. Introduction

TB is a contagious disease and ranks as the second leading cause of death globally from a single infectious agent [1]. In 2023, there were 10.8 million new cases of TB diagnosed globally, along with 1.25 million deaths related to TB infection [1]. TB diagnosis and treatment have become disrupted due to the COVID-19 pandemic worldwide [2]. Hence, there will be an increase in estimated TB incidence in the upcoming years [1]. Identifying TB at an early stage is essential for accomplishing the global goals outlined in the END TB strategy [3]. Early diagnosis and treatment initiation aid in preventing the community from spreading the disease and lead to better patient outcomes. Newer diagnostic methods with high sensitivity and specificity for active tuberculosis (ATB) at an early stage are the need of the hour that can be implemented in point-of-care settings.

Sputum microscopy to identify Acid-fast bacilli (AFB) is a conventional method that is widely available and cost-effective and is currently used as a point-of-care diagnostic method. However, it has limited sensitivity and cannot distinguish between live and dead bacilli or identify drug resistance [4]. It also cannot differentiate *Mycobacterium tuberculosis* (MTB) from other atypical mycobacteria. Mycobacterial culture is recognized as the benchmark method, especially in extra-pulmonary and pauci-bacillary samples, but it takes a few weeks for the report to be available [5]. Introduction of Nucleic Acid Amplification Tests (NAAT) like Gene Xpert MTB/RIF and Gene Xpert MTB/RIF Ultra has revolutionized the diagnosis of TB worldwide. NAAT-based tests are rapid, have high sensitivity and specificity and are beneficial in the diagnosis of TB in Extra-pulmonary and pauci-bacillary samples [6]. The pooled sensitivity and specificity of these conventional methods in diagnosing PTB are 61% and 98% for sputum microscopy, 99% and 100% for Mycobacteria liquid culture and 92% and 99% for Xpert MTB/RIF [7]. But these are expensive, require specific infrastructure and trained personnel, and cannot be made available in resource-limited settings.

There is a significant gap between the diagnosis of TB and the identification of individuals with active TB disease. This mandates the development of accurate, rapid, cost-effective, and unsophisticated diagnostic methods at point-of-care facilities. Identifying and using biomarkers from non-invasively available samples like sputum, blood and urine are the current scope of research in TB diagnosis. A biomarker is described as a ‘specific characteristic’ that is measured to indicate normal biological processes, disease processes, or responses to an exposure or treatment [8]. There are two types of biomarkers that are being widely studied in the realm of TB. They are pathogen-based biomarkers like antigen and DNA detection and host-based biomarkers like antibodies, cytokines and chemokines, RNA, and other proteins. IL-2 is a cytokine generated by T-cells that is activated by antigens, and its levels are notably increased in response to MTB infection [9]. It plays an important role in inducing T-cell proliferation, generating cell-mediated immune responses, and contributing to granuloma formation in TB disease. Additionally, an elevated expression of IL-2 in response to MTB-specific antigens has been documented in earlier research. Studies have shown that IL-2 is also an effective marker in differentiating Latent TB Infection (LTBI) and ATB infection [10]. LAM is an essential element of the mycobacterial cell wall and is also a critical virulence factor [11].

LAM is released into circulation and can be detected in body fluids like urine and serum. WHO recommends the utilization of Lateral Flow-LAM assay for diagnosing TB in Patients Living with HIV [12]. Detection of LAM in serum has been shown to be a reliable marker of PTB and EPTB [13]. Micro-RNAs are short, non-coding RNA molecules that mainly modulate gene expression after transcription. The expression of miR-29a is shown to have elevated in the serum of patients with TB [14]. Therefore, we carried out this study to assess the expression of IL-2, LAM and miR-29a in active PTB and EPTB.

## 2. Materials and methods

### 2.1. Ethical approval

This study received approval from the Institute Human Ethics Committee (Ref No: IHEC-II/0322/23, dated 23.03.2023) at Chettinad Hospital and Research Institute, Chettinad Academy of Research and Education. Written informed consent was obtained from all participants before their enrolment in the study. The study protocol and data collection for this investigation adhere to the Declaration of Helsinki ethical principles for medical research involving human subjects.

### 2.2. Study population and sub-groups

This prospective study enrolled a total of 46 patients between May 2023 and December 2023. Participants were recruited from the Outpatient Department (OPD) of Respiratory Medicine at Chettinad Hospital and Research Institute. All the participants belong to the South Indian population of Asian Ethnicity. They were subjected to a thorough physical examination and a proforma-based interview to collect demographic data and medical history. Details of diagnosis, sputum investigations reports (AFB smear and NAAT) and tissue/fluid reports (histopathology and NAAT), along with baseline blood workup like Complete Blood Counts, Renal function test, liver function test, HIV and hepatitis serology, and Chest X-ray were collected from their medical records. Participants were recruited under three sub-groups - PTB group, EPTB group, and Control group. Patient details are provided in Table 1.

**Table 1.** Demographic data of the study population.

Category	All participants (*N* = 46)	PTB group (*N* = 17)	EPTB group (*N* = 13)	Control group (*N* = 16)
Gender				
Male	27 (58.7%)	13 (76.5%)	6 (46.2%)	8 (50.0%)
Female	19 (41.3%)	4 (23.5%)	7 (53.8%)	8 (50.0%)
Age				
Mean (years)	49.6	46.9	41.9	58.6
Range (years)	17-81	17-76	18-65	22-81
Ethnicity				
Indian	46	17	13	16
Symptoms				
Cough	40 (85.1%)	17 (100%)	7 (53.8%)	16 (100%)
Breathlessness	15 (31.9%)	4 (23.5%)	3 (23.1%)	8 (50.0%)
Fever	26 (55.3%)	15 (88.2%)	9 (69.2%)	2 (12.5%)
Loss of appetite	24 (51.1%)	15 (88.2%)	7 (53.8%)	2 (12.5%)
Loss of weight	24 (51.1%)	15 (88.2%)	7 (53.8%)	2 (12.5%)
Others	13 (27.7%)	1 (5.9%)	11 (84.6%)	1 (6.25%)

For the PTB group, newly diagnosed micro-biologically confirmed PTB patients were enrolled before initiation of Anti-Tubercular Treatment (ATT). For the EPTB group, newly diagnosed micro-biologically/clinically diagnosed EPTB patients were recruited before initiation of ATT. In both the group’s patients who had a prior history of ATT, history of TB, co-morbidities like diabetes, chronic liver disease, chronic kidney disease, immunosuppression or in an immuno-compromised state were excluded.

For the control group, patients with stable non-infectious respiratory disease like Chronic Obstructive Pulmonary Disorder (COPD), Bronchial Asthma (BA), and Interstitial Lung Disease- Idiopathic Pulmonary Fibrosis (IPF) were studied. Chest X-ray was taken to rule out ATB and Tuberculin Skin Test (TST) was done. Those with positive results for TB infection in TST were excluded. Participants with a prior history of ATT, past history of TB, co-morbidities like diabetes, chronic kidney disease, chronic liver disease, and immunosuppression or in an immuno-compromised state were also excluded.

### 2.3. Laboratory procedures

Blood samples were collected from participants before the initiation of ATT, enrollment and informed consent. Vacutainer blood collection tubes (with clot activator) were used, and 5 ml of venous blood was collected from each participant under strict aseptic precautions. Samples were transported to the lab within 15–20 min in an ice box (6–8 °C). The serum was isolated from the blood using a cooling centrifuge (4–8 °C) at 2000 RPM for 10 min. Serum was pipetted and transferred into cryovials at aliquots of 0.5 ml, labeled and stored at −80 °C.

#### 2.3.1. Measurements of IL-2 by ELISA

IL-2 was measured in serum from 13 controls, 13 PTB, and 12 EPTB patients. The ELISA was used for the assessment of serum levels of IL-2 was assessed by using EliKine^TM^ Human IL-2 ELISA Kit (Catalog No.- KTE6014Atlanta 30303, Georgia, USA). A total of 100 μL of diluted standard or sample was added to each well in duplicate. The plate was covered and incubated at room temperature for 2 h. After incubation, the liquid was removed, and the wells were washed three times with 250 μL of 1X wash buffer, ensuring complete removal. Then, 100 μL of diluted 1X Human IL-2 Detection Antibody was added and incubated for 1 h, followed by another wash. Next, 100 μL of 1× Streptavidin-HRP was added, incubated for 30 min, and washed five times. Subsequently, 100 μL of HRP Substrate was added, incubated for 15 min, and 50 μL of stop solution was introduced. Finally, absorbance was measured at 450 nm within 30 min. Serum concentration of IL-2 was mentioned as a pg/mL unit.

#### 2.3.2. Measurement of LAM by ELISA

LAM was measured in serum from 13 controls, 13 PTB, and 12 EPTB patients. The ELISA was used for the assessment of serum levels of LAM using Lipoarabinomannan (LAM) ELISA Kit (Catalog No: abx576780, Abbexa-Cambridge, CB4 0GJ, United Kingdom). A total of 100 μL of diluted standard or sample was added to each well in duplicate, covered, and incubated at room temperature for 2 h. The liquid was then removed, and wells were washed three times with 350 μL of 1X wash buffer. Next, 100 μL of reagent A was added, incubated for 1 h, and washed again. This was followed by adding 100 μL of reagent B, incubating for 30 min, and washing five times. Finally, 90 μL of TMB Substrate was added, incubated for 20 min at 37 °C, followed by 50 μL of stop solution, and absorbance was measured at 450 nm immediately. Serum concentration of LAM was mentioned as a pg/mL unit.

#### 2.3.3. RNA extraction and real-time PCR

**Figure 1.**
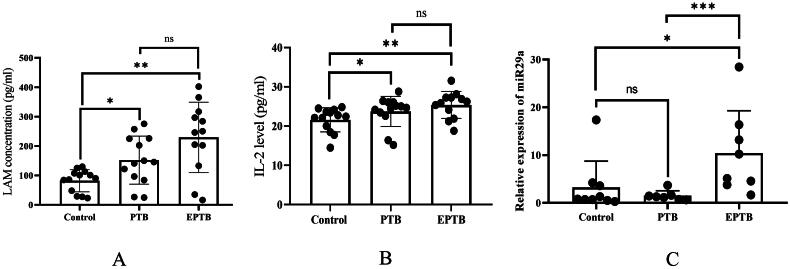
Expression of LAM, IL-2, and miR-29a in control, PTB, and EPTB groups. (A) Expression of LAM (pg/ml) in the control, pulmonary tuberculosis (PTB), and extra-pulmonary tuberculosis (EPTB) groups. The *p*-values for PTB and EPTB compared to the control were 0.0256 and 0.002, respectively, while PTB vs. EPTB was 0.07, (B) Serum IL-2 levels in the control, PTB, and EPTB groups. The *p*-values for PTB and EPTB compared to the control were 0.0197 and 0.0083, respectively, while PTB vs. EPTB was 0.230, (C) Relative expression of miR-29a in PTB and EPTB patients. The *p*-values for PTB and EPTB compared to the control were 0.7577 and 0.0111, respectively, while PTB vs. EPTB was <0.001. Bars represent the mean ± SD. Statistical comparisons were conducted using the Mann-Whitney U test. Statistical significance is indicated as follows: *p < 0.05, **p < 0.01, ***p < 0.001.

Total RNA was isolated from normal control group, PTB, and EPTB group patient’s serum samples using RNAZol reagent (Sigma-Aldrich, St. Louis, MO, USA) using the manufacturer’s protocol. To check miRNA expression, the manufacturer’s instructions were followed to reverse transcription of miR-29a using the Thermoscript RT system reagent (Assay Name- hsa-miR-29a, Assay ID- 002112, mature miRNA sequence- UAGCACCAUCUGAAAUCGGUUA, Catalog No- 4427975, Invitrogen, CA, USA). Quantitative real-time PCR (q-RT-PCR) was carried out to quantify miR-29a levels in all the serum samples. To quantify miR-29a levels from the serum samples, quantitative real-time PCR (q-RT-PCR) was carried out using the Applied Biosystems^®^ QuantStudio5 Real-Time PCR System, employing TaqMan^™^ primers specific to miR-29a and TaqMan^™^ Universal Master Mix II, no UNG (Catalog number- 4440043, Applied Biosystems). RNU6b (Assay Name- RNU6B, Assay ID- 001093, Catalog No- 4427975, Primer sequence- CGCAAGGATGACACGCAAATTCGTGAAGCGTTCCATATTTTT) served as the endogenous control for the miRNA expression analysis [15]. miR-29a expression was analyzed in serum samples from 24 patients, including 9 controls, 7 PTB patients, and 8 EPTB patients. The results are represented in Figure 1C, with each individual data point.

#### 2.3.4. Receiver operating characteristic (ROC) curve analysis

We conducted a validation analysis of LAM and IL-2 levels measured using ELISA and miR-29a expression levels measured using qRT-PCR in patients from the control, PTB, and EPTB groups. The ROC curve was generated by plotting sensitivity (%) (true positive rate) against (100% – specificity %) (false positive rate) across various threshold settings. Discriminatory accuracy was assessed using the area under the ROC curve (AUC).

#### 2.3.5. Statistical analysis

All data were collected from distinct sample groups, including the control group (healthy individuals), the PTB patients group, and the EPTB patients group. Statistical analysis was performed using GraphPad Prism version 8.0. The non-parametric Mann–Whitney *U* test was used to evaluate statistical significance. A *p*-value of *p < 0.05, **p < 0.01, and ***p < 0.001 was considered statistically significant.

## 3. Results

### 3.1. Clinical relevance of the study population

The clinical study involved 46 participants, 17 in the PTB group, 13 in the EPTB group and 16 in the control group. Demographic data broken down by subgroups of the participants is presented in Table 1. The study population consisted of 27 (58.7%) male and 19 (41.3%) female participants. The mean age was 49.6 years. The most common reported symptom was cough, followed by fever, loss of appetite, and loss of weight. Among the tested samples, 30 out of 46 patients were diagnosed with active tuberculosis; among them, 17 patients (36.95%) had PTB, and 13 patients (28.26%) had EPTB.

In the PTB group, the diagnosis of all 17 participants involved chest X-rays, sputum Acid Fast Bacilli (AFB) smears, as well as sputum Gene Xpert MTB/RIF and sputum First Line Probe Assay (LPA), following the guidelines of the National Tuberculosis Elimination Programme (NTEP). In the EPTB group, 6 of them had tubercular pleural effusion, 5 had tubercular cervical lymphadenitis and 2 had ileocecal tuberculosis. None among the EPTB group had concurrent active PTB. The diagnostic details of the EPTB group are presented in Table 2. Fluid ADA (5/13), tissue HPE, Fine Needle Aspiration Cytology (FNAC), and Gene Xpert MTB/RIF were the diagnostic methods utilized to diagnose EPTB. In both groups, among those who had a microbiological diagnosis, none of them had Drug-Resistant TB (DR-TB). None of them had disseminated or miliary TB. In the control group, nine of the participants were COPD, three were Bronchial Asthma, and four were ILD-IPF patients. Baseline blood investigation values, including complete blood count, renal function tests, liver function tests, random blood sugar, and viral serology, were obtained from the participants’ medical records, and with notable results shown in Table 3 [16]. None of them were positive for HIV or Hepatitis viral serology tests.

**Table 2.** Diagnostic details of EPTB group.

Diagnostic test	Numbers
Tissue biopsy followed by Histopathological Examination (HPE) suggestive of active TB	10 (6 pleural + 2 Lymph node+ 2 ileocecal)
Tissue Biopsy followed by Gene Xpert MTB/RIF	10 (MTB detected in 2)
Fine Needle Aspiration Cytology (FNAC) suggestive active TB	3 (Lymph node)
Fine Needle Aspiration Cytology (FNAC) followed by Gene Xpert MTB/RIF	3 (MTB detected-3)

**Table 3.** Significant baseline blood investigations of the participants.

Parameter	Mean value					
	PTB group		EPTB group		Control group	
	Male	Female	Male	Female	Male	Female
Hemoglobin (g/dL)	11.2	11.0	11.5	11.2	12.3	12.0
Total Protein (g/dL)	7.1	7.8	7.3	7.2	7.0	6.9
Albumin (g/dL)	3.2	3.4	3.6	3.4	3.9	3.8

^*^ Reference ranges for Indian population: [16].

Hemoglobin: Males: 12.3–16.4 g/dL; Total protein: Males: 6.4–8.0 g/dL; Albumin: Males: 3.5–4.9 g/dL; Females: 11.0–14.4 g/dL; Females: 6.3–8.0 g/dL; Females: 3.4–4.8 g/dL.

### 3.2. Analysis of serum lipoarabinomannan (LAM) in control and tuberculosis patients

Figure 1A demonstrates a significant increase in LAM levels in both the PTB (*p* = 0.0256) and EPTB (*p* = 0.002) groups compared to the control (TB-negative) group. Additionally, a notable difference was observed between the PTB and EPTB groups (Figure 1A). However, this difference was not statistically significant (*p* = 0.070).

### 3.3. Analysis of serum interleukin 2 (IL-2) in control and tuberculosis patients

An ELISA assay was performed to check the IL-2 level in the serum of control and TB patients. Figure 1B displays a significant upregulation in the IL-2 level in the PTB (*p* = 0.0197) and EPTB (*p* = 0.0083) groups compared to the control group. Whereas, among the PTB and EPTB groups, no significant difference was observed (*p* = 0.230).

### 3.4. The expression level of miR-29a in the serum samples of control and tuberculosis patients

q-RT-PCR was done to check the miR-29a expression from the serum samples of the control group patients and TB patients of both the PTB and EPTB groups. Here, the result shows a significant upregulation of miR-29a expression in EPTB (*p* = 0.0111) compared to the control group, whereas a non-significant change was found in the PTB (*p* = 0.7577) group compared to the control group (Figure 1C). However, a significant change was observed between the PTB and EPTB groups (*p <* 0.001).

### 3.5. Receiver operating characteristics (ROC) curve analysis

**Figure 2.**
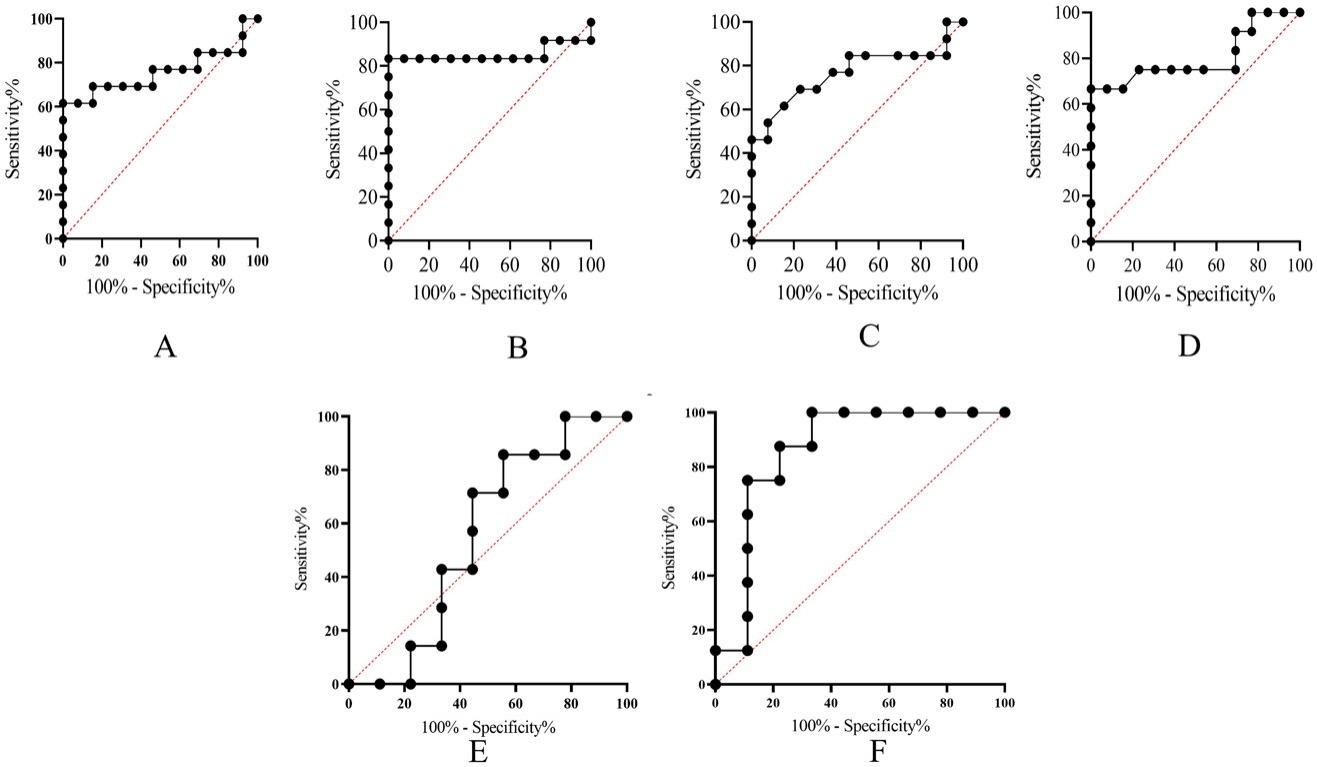
ROC curve analysis for assessing the diagnostic potential of LAM, IL-2, and miR-29a. (A, B) ROC analysis for LAM: the AUC for PTB vs. control was 0.7574 (95% CI: 0.5528–0.9620, *p* = 0.0257), and for EPTB vs. control, 0.8526 (95% CI: 0.6623–1.000, *p* = 0.0028). (C, D) ROC analysis for IL-2: the AUC for PTB vs. control was 0.7663 (95% CI: 0.5708–0.9617, *p* = 0.0210), and for EPTB vs. control, 0.8045 (95% CI: 0.6156–0.9934, *p* = 0.0098). (E, F) ROC analysis for miR-29a: the AUC for PTB vs. control was 0.5556 (95% CI: 0.2623–0.8488, *p* = 0.7110), and for EPTB vs. control, 0.8611 (95% CI: 0.6667–1.000, *p* = 0.0124).

Receiver operating characteristic (ROC) analysis was performed to assess the diagnostic potential of serum LAM, IL-2, and circulating miR-29a in distinguishing PTB and EPTB patients from control group patients. Expression data were used to generate ROC curves for PTB vs. control and EPTB vs. control groups. As shown in (Figure 2A,B), the area under the curve (AUC) for LAM was 0.7574 (PTB vs. control) and 0.8526 (EPTB vs. control), indicating a moderate discriminatory ability. The sensitivity and specificity for PTB were approximately 63%, while for EPTB; they were 69% and 67%, respectively. Similarly, (Figure 2C,D) depict the ROC analysis for IL-2, with an AUC of 0.7663 for PTB vs. control and 0.8045 for EPTB vs. control. The sensitivity for PTB and EPTB was 64.61% and 67.85%, with corresponding specificities of 61.15% and 64.10%, suggesting a moderate diagnostic performance. For miR-29a (Figure 2E,F), the AUC was 0.5556 for PTB vs. control and 0.8611 for EPTB vs. control. While miR-29a showed limited discriminatory ability between PTB and controls (sensitivity and specificity ∼53%; 95% CI: 0.2623–0.8488, *p* = 0.7110), it demonstrated potential as a biomarker for differentiating EPTB, with a sensitivity of 70.31% and specificity of 68.06% (95% CI: 0.6667–1.000, *p* = 0.0124).

## 4. Discussion

The WHO launched “The End TB Strategy” in 2015, aiming to reduce TB incidence by 90% and mortality by 95% by 2035 [2]. A key challenge in achieving this goal is the large number of undiagnosed TB cases. To address this, research and innovation in TB diagnostics are crucial [2]. Developing new diagnostic tools, particularly point-of-care tests, is essential for early detection and timely treatment, especially in resource-limited settings. While biomarker-based diagnostic tests have been extensively studied, apart from MTB DNA-based tests (CBNAAT, LPA) and LAM detection in PLHIV, no universally reliable tool has emerged due to heterogeneity in research findings. Variations in TB pathogenesis, disease manifestation, and MTB virulence across populations complicate the identification of a single diagnostic marker. This study evaluated IL-2, LAM, and miR-29a as potential diagnostic biomarkers for PTB and EPTB. To ensure uniformity, only immunocompetent individuals without comorbidities were recruited. All participants underwent CBNAAT for rifampicin resistance testing, aligning with the Universal Drug Susceptibility Testing (UDST) protocol. Blood samples were collected before treatment initiation to minimize ATT-induced immunological changes. Baseline investigations, including viral serology, ruled out co-infections. TB patients exhibited anemia and hypoalbuminemia (Table 3), consistent with previous studies [17–19], likely due to malnutrition and chronic inflammation. Identifying novel biomarkers alongside conventional diagnostics may enhance early TB detection, reducing mortality. MTB influences host cytokine production, and cytokine profiling can distinguish latent from active TB [20,21]. Upon TB infection, cytokines such as IL-2, IL-12, IL-17, IFN-γ, and TNF-α are released [22,23], playing a role in immune defense. IL-2 and IFN-γ are widely studied for TB diagnosis [24,25]. While the Interferon Gamma Release Assay (IGRA) is commonly used, it cannot differentiate latent from active TB, similar to the tuberculin skin test [26,27]. Recent studies suggest IL-2 as a promising diagnostic marker when used in combination [27]. IL-2, an important cytokine, plays a crucial role in inducing T-cell proliferation, generating cell-mediated immune responses, and contributing to granuloma formation in TB disease. Additionally, an elevated expression of IL-2 in response to MTB-specific antigens has been documented in earlier research. Many studies have revealed that after the MTB infection, the IL-2 level was found to be high [28,29]. In this study, serum LAM and IL-2 levels were analyzed for TB diagnosis. LAM, a key MTB cell wall component, is crucial in TB pathogenesis and immune modulation. It is detectable in serum and urine, making it a potential biomarker [30,31]. Serum LAM and IL-2 levels were significantly elevated in PTB and EPTB groups compared to controls (Figure 1A,B), indicating their association with TB severity. Circulating miRNAs are emerging as predictive biomarkers capable of distinguishing active from latent TB [32,33]. These small non-coding RNAs regulate gene expression and play roles in immune responses [34,35]. Dysregulated miRNA expression is linked to disease progression [36,37], including TB pathogenesis [38]. MTB modulates miRNA expression, affecting immune cells like T cells, NK cells, dendritic cells, and macrophages [38]. miRNAs have been reported to contribute in lung homeostasis, and their altered expression is associated with various lung diseases [39–41]. This study analyzed serum miR-29a levels in PTB, EPTB, and control groups. MiR-29a is involved in adaptive immunity and immune regulation [42]. Studies have reported its elevated expression in TB patients, where it suppresses IFN-γ and modulates host immune responses [43]. One meta-analysis study reported that miR-29a has diagnostic performance as a biomarker of ATB diagnosis [44]. Additionally, Garg et al. (2021) demonstrated significantly higher miR-29a levels in TB patients, supporting its diagnostic potential [14]. Consistently, our study found significantly higher miR-29a expression in EPTB patients compared to the control group, while PTB patients showed non-significant upregulation. Further­more, to evaluate the diagnostic potential of LAM, IL-2, and miR-29a, ROC curve analysis was conducted. As shown in (Figure 2A,B), LAM, and in (Figure 2C,D), IL-2 demonstrated moderate to strong diagnostic accuracy in distinguishing PTB and EPTB from control individuals, with better sensitivity and specificity for EPTB. This may reflect variations in immune response between pulmonary and extra-pulmonary tuberculosis. In contrast, miR-29a showed significant potential for EPTB (AUC = 0.8611, *p* = 0.0124) (Figure 2F) but had limited discriminatory power for PTB (AUC = 0.5556, *p* = 0.7110) (Figure 2E), suggesting its expression is possibly more relevant to extra-pulmonary disease and may correlate with TB severity [45]. Further validation in larger cohorts is necessary to confirm the clinical applicability of these biomarkers, particularly for EPTB. Recent advancements in TB diagnosis emphasize biomarker combinations to improve screening accuracy, particularly over the conventional tests like TST and IGRA. Ongoing research explores interactions between miRNAs, cytokines, and LAM to validate their roles in TB pathogenesis. Our findings highlight IL-2, LAM, and miR-29a as promising diagnostic markers for differentiating PTB from EPTB, supporting their potential use in early TB detection [46,47].

## Conclusion

Despite advanced TB diagnostic strategies, accurate diagnosis remains challenging. Detecting LAM and IL-2 in serum offers a promising diagnostic approach for PTB and EPTB patients, whereas miR-29a provides a promising approach for diagnosing EPTB patients. However, validation in larger cohorts across diverse populations is needed to confirm the use of these biomarkers in future.

## Data Availability

The data supporting the findings of this study are available from the corresponding authors, AB, SP, and SR, upon reasonable request.

## Limitations

In this study, individuals with latent TB infection (determined by TST) were excluded, and a separate subgroup with latent TB was not included. Therefore, the expression of the analyzed biomarkers in this population remains unexamined. Additionally, the small sample size limits the statistical strength of our findings, preventing definitive clinical conclusions. However, as a pilot study, our findings suggest that LAM, IL-2, and circulating miR-29a may have potential in TB diagnosis. Further studies with larger cohorts are needed to validate the clinical reliability and diagnostic accuracy of these markers before their potential use in TB diagnosis.
